# Ferrous iron content of intravenous iron formulations

**DOI:** 10.1007/s10534-016-9923-7

**Published:** 2016-03-08

**Authors:** Ajay Gupta, Raymond D. Pratt, Alvin L. Crumbliss

**Affiliations:** Division of Nephrology, School of Medicine, University of California Irvine, 101 The City Drive South, City Tower, Suite 400, Orange, CA 92868-3217 USA; Rockwell Medical, Inc, Wixom, MI USA; Department of Chemistry, Duke University, Durham, NC USA

**Keywords:** Chronic hemodialysis, Ferric iron, Ferrous iron, Intravenous iron, Iron supplementation

## Abstract

The observed biological differences in safety and efficacy of intravenous (IV) iron formulations are attributable to physicochemical differences. In addition to differences in carbohydrate shell, polarographic signatures due to ferric iron [Fe(III)] and ferrous iron [Fe(II)] differ among IV iron formulations. Intravenous iron contains Fe(II) and releases labile iron in the circulation. Fe(II) generates toxic free radicals and reactive oxygen species and binds to bacterial siderophores and other in vivo sequestering agents. To evaluate whether differences in Fe(II) content may account for some observed biological differences between IV iron formulations, samples from multiple lots of various IV iron formulations were dissolved in 12 M concentrated HCl to dissociate and release all iron and then diluted with water to achieve 0.1 M HCl concentration. Fe(II) was then directly measured using ferrozine reagent and ultraviolet spectroscopy at 562 nm. Total iron content was measured by adding an excess of ascorbic acid to reduce Fe(III) to Fe(II), and Fe(II) was then measured by ferrozine assay. The Fe(II) concentration as a proportion of total iron content [Fe(III) + Fe(II)] in different lots of IV iron formulations was as follows: iron gluconate, 1.4 and 1.8 %; ferumoxytol, 0.26 %; ferric carboxymaltose, 1.4 %; iron dextran, 0.8 %; and iron sucrose, 10.2, 15.5, and 11.0 % (average, 12.2 %). The average Fe(II) content in iron sucrose was, therefore, ≥7.5-fold higher than in the other IV iron formulations. Further studies are needed to investigate the relationship between Fe(II) content and increased risk of oxidative stress and infections with iron sucrose.

## Introduction

All formulations of iron suitable for intravenous (IV) administration are colloidal suspensions of iron oxide nanoparticles, with particle size ranging from 5 to 40 nm. These nanoparticles are composed of a polynuclear iron-oxyhydroxide/oxide central core surrounded by a carbohydrate shell, which stabilizes the core and protects the nanoparticles against further polymerization (Neiser et al. 2015). Different IV iron formulations have distinct properties and display a wide range of stability, degradation kinetics in the circulation, and antigenic potential, depending on the type of stabilizing carbohydrate (Geisser and Burckhardt 2011).

The core particle size has an influence on iron lability: the smaller the size, the greater the surface area-to-volume ratio and, therefore, the greater the lability of bound iron. This is due to the physical dissociation of iron from the particle surface. The electrovalence of iron also influences iron lability. Ferrous iron [Fe(II)] is kinetically more reactive (labile) than ferric iron [Fe(III)] with respect to exchanging its nearest neighbor bonding atoms (ligands) (Helm and Merbach 2005). This means that dissociation from the nano-particle surface is more facile for Fe(II). However, this also means that its reaction with potential in vivo binding sites is less discriminating, thus making delivery to the desired transferrin site less probable or at least delayed. Fe(II) can readily bind to bacterial siderophores, potentially leading to infection (Farkas et al. 2001). In addition Fe(II) can readily undergo redox reactions with metabolites, such as peroxides, to generate highly toxic reactive oxygen species, which cause oxidative stress and damage to cellular constituents (Crichton and Boelaert 2001; Geisser and Burckhardt 2011).

It has become increasingly recognized that the size and variability of the iron core and the types of impurities that are present (e.g., the ratio of divalent and trivalent iron) are among the quality attributes of IV iron formulations that impact efficacy and safety (European Medicines Agency 2013). Therefore, we performed comparative analyses of the Fe(II) content of different formulations of IV iron that are available for commercial use in the United States.

## Methods

Commercially available IV iron formulations were obtained and assayed for Fe(II) and total iron ≤5 min after the vials were opened. All measurements of Fe(II) and total iron were performed in quadruplicate, taking samples from the dilute iron solution.

### Fe(II) iron content

A 100 µL volume of sample was mixed with 1 mL of 12 M concentrated HCl (37 % HCl by weight) to produce clear yellow solutions (1/11 dilution) of each IV iron formulation. A 100 µL volume of the (1/11) dilution was mixed with 10 mL of H_2_O to achieve a 1/1100 dilution (0.1 M HCl concentration). A 200 µL volume of 1/1100 dilution was mixed with 50 µL of 0.7 M sodium acetate to adjust the pH to 4.0 after which 20 µL of 1 mg/mL ferrozine was added. After a 5-min incubation, this solution was scanned on the ultraviolet (UV) plate reader to determine the amplitude of the peak at 562 nm wavelength. Fe(II) content was determined by reference to a 5 µM standard. The concentration of the Fe(II) in the initial sample was calculated, after adjustment for dilutions, and reported as the percent of total iron. The percent of total iron was compared to the label value for each of the IV iron preparations.

### Total iron content

The 1/1100 dilution was further diluted to a final HCl concentration of 0.1 M. A 100 µL volume was mixed with 1.9 mL of 12 M HCl to achieve a 1/22,000 dilution. A 200 µL volume of the 1/22,000 dilution was mixed with 20 µL of 10 mg/mL ascorbic acid to convert all iron to Fe(II). After adjusting the pH to 4.0 with 50 µL of sodium acetate, 20 µL of ferrozine was added. The 562 nm peak was determined after a 5-min incubation, and the total iron content of the sample was calculated by reference to the Fe(II) iron standard.

## Results

The Fe(II) content was measured in single lots of iron dextran (INFeD™), ferumoxytol (Feraheme™), and ferric carboxymaltose (Injectafer™); 2 lots of iron gluconate (Ferrlecit™); and 3 lots of iron sucrose (Venofer™) (Table 1). As a fraction of the measured total iron, the measured Fe(II) content was <1 % for iron dextran and ferumoxytol, 1–2 % for ferric carboxymaltose and iron gluconate, and 10–15 % for iron sucrose. The Fe(II) content in iron sucrose was ≥7.5-fold higher than in any of the other IV iron formulations.

**Table 1:** Fe(II) and total iron content of intravenous iron formulations

Iron formulation	Lot number Expiry date	Percentage Fe(II) iron	Total iron	
			Measured value (mg/mL)	Labeled value (mg/mL)
Iron dextran (InFed™)	14W21A Oct 2017	0.8	49	50
Ferumoxytol (Feraheme™)	ACO386 Feb 2019	0.26	30	30
Ferric carboxymaltose (Injectafer™)	1501901 Feb 2017	1.4	48	50
Iron gluconate (Ferrlecit™)	A4040 Nov 2017	1.4	12.7	12.5
	A5063 April 2018	1.8	–^a^	–^a^
	Average	1.6		
Iron sucrose (Venofer™)	4351A Nov 2016	10.2	19.4	20
	5163 May 2017	15.5	–^a^	–^a^
	5127A Mar 2017	11.0	–^a^	–^a^
	Average	12.2		

^a^ Total iron content was not determined for these lot numbers because analysis of total iron content for the first lot and for the other iron formulations generated label values

## Discussion

The differences in Fe(II) content between the various IV iron formulations that we observed are consistent with the differences in polarographic signatures due to the Fe(III)/Fe(II) couple between the different IV formulations (Neiser et al. 2015). In this study, we found that the Fe(II) content in iron sucrose (Venofer™), the most commonly used IV iron formulation in hemodialysis patients in the United States, was significantly higher than that in the other IV formulations that we evaluated.

Intravenous iron formulations release 2–6 % of their total iron into the circulation whereas the majority of iron undergoes uptake by the reticuloendothelial macrophages in liver, spleen, lymph nodes, and bone marrow (Beshara et al. 1999). An increase in Fe(II) in the circulation with iron sucrose would be expected to enhance generation of free radicals and oxidant stress relative to other IV iron formulations. Stefansson et al. (2011) measured plasma iron and oxidative stress parameters before and 10 min after IV injection of 100 mg iron sucrose or iron dextran in 20 chronic hemodialysis patients and found that non-transferrin-bound iron increased significantly more after administration of iron sucrose than after administration of iron dextran (86 ± 42 vs 45 ± 45 %, respectively; P < 0.05). Furthermore, plasma ascorbic free radical did not change after administration of iron dextran (−1.8 ± 11.2 %) but increased by 29 ± 31.3 % after administration of iron sucrose (P < 0.01). Additionally, protein carbonyls increased after administration of iron sucrose (P < 0.05) but not after administration of iron dextran. Wang et al. (2008) demonstrated increased oxidation of ascorbic acid in human plasma and whole blood with iron sucrose relative to iron gluconate. All 3 biomarkers (non-transferrin-bound iron, protein carbonyl, and ascorbate radical levels) suggest a higher level of oxidative stress associated with iron sucrose administration.

Release of non-transferrin bound iron following treatment of hemodialysis patients with iron sucrose has also been associated with enhanced growth of *Staphylococcus aureus* after inoculation into serum samples from these patients (Barton Pai et al. 2006). Iron sucrose also has specific immunologic effects on monocytes and hematopoietic stem cells that were not induced by the other IV iron preparations (Fell et al. 2014). Iron sucrose activated monocyte subsets leading to significantly increased CD86 expression. Simultaneously, CD16 and CX3CR1 expression and monocytic phagocytosis capacity were decreased. Additionally, differentiation of monocytes from hematopoietic CD34+ stem cells was almost completely abolished after stimulation with iron sucrose. This effect of iron sucrose on immune cells may contribute to a higher risk of infections observed in dialysis patients who are treated with iron sucrose (Brookhart et al. 2016; Sirken et al. 2006).

Our study did not measure Fe(II) after addition of various iron compounds to plasma or blood or following administration to patients because divalent iron is rapidly oxidized in the plasma to Fe(III) by plasma ceruloplasmin (Roeser et al. 1970). Furthermore, the results of our study should be considered with caution because we have not studied the fate of Fe(II) and Fe(III) in the plasma with respect to transferrin binding or uptake by parenchymal cells and macrophages. Other studies, as referenced above, have noted the difference in IV administration of iron sucrose with respect to infection, immune response, and oxidative stress. Results presented here suggest this may be due, at least in part, to high Fe(II) content. Consequently, the significantly higher Fe(II) content in iron sucrose (Venofer™) is a cause for concern given the toxic nature of this species. Further studies are needed to investigate the relationship between Fe(II) content and increased risk of oxidative stress and infections with iron sucrose.
